# Continuous Infusion Pressure Measurement in Predefined Interfascial and Intramuscular Tissue Conditions: An Ex Vivo Pilot Study

**DOI:** 10.3390/jcm15093301

**Published:** 2026-04-26

**Authors:** Mateusz Wilk, Karol Jedrasiak, Aleksandra Suwalska, Marek Gzik, Piotr Wodarski

**Affiliations:** 1 Collegium Medicum, WSB University, 41-300 Dabrowa Gornicza, Poland; 2 Department of Anesthesiology and Intensive Care, University Clinical Center, Medyków 14, 40-055 Katowice, Poland; 3 Department of Transport and Computer Science, WSB University, 41-300 Dabrowa Gornicza, Poland; 4 Department of Data Science and Engineering, Faculty of Automatic Control, Electronics and Computer Science, Silesian University of Technology, 44-100 Gliwice, Poland; 5 Department of Biomechatronics, Faculty of Biomedical Engineering, Silesian University of Technology, 44-100 Gliwice, Poland

**Keywords:** nerve block, catheter, pressure, infusion, regional anesthesia

## Abstract

**Background:** Perineural catheter migration is a clinically relevant cause of continuous block failure, but the present study was not designed to model true clinical displacement. Instead, we investigated whether low-rate infusion pressure differs between two predefined catheter–tissue environments, interfascial and intramuscular, under controlled ex vivo conditions. **Methods:** Sixty porcine thigh specimens were studied. Under ultrasound guidance, a catheter-over-needle system with a multi-orifice catheter was placed either in the interfascial plane or intramuscularly, with one measurement obtained from each specimen. After baseline recording outside the tissue, saline was infused at 5 mL/h for 10 min. Pressure recordings were normalized to baseline. For each trace, a representative value was obtained using a predefined automated stable-segment algorithm, and between-group differences were assessed using Welch’s *t*-test. **Results:** Mean normalized pressure was higher during intramuscular than interfascial infusion (0.3346 ± 0.0635 PSI [17.3 ± 3.3 mmHg] vs. 0.1917 ± 0.0285 PSI [9.9 ± 1.5 mmHg]). The between-group difference was significant (mean difference: 0.1430 PSI [7.4 mmHg], 95% CI: 0.1181 to 0.1679 PSI; p = 7.22 × 10^−15^), with a very large standardized effect size (Hedges’ g = 2.87), reflecting strong statistical separation between the two predefined groups under controlled ex vivo conditions rather than clinical discriminative ability. However, the absolute pressure difference remained small. **Conclusions:** Under controlled ex vivo conditions, mean normalized infusion pressure differed between predefined interfascial and intramuscular catheter positions. However, the absolute difference was small. This binary model does not represent real catheter displacement, and the findings do not support current clinical applicability, individual-level interpretation, or the definition of a clinically usable threshold. The results should be considered exploratory and hypothesis-generating.

## 1. Introduction

Continuous nerve and plexus blocks offer a good alternative to repeated single-shot blocks. They provide analgesia comparable to that of an epidural block, but without the latter’s drawbacks (e.g., hypotension, urinary retention, pruritus, and restricted mobility) [1,2]. Continuous peripheral nerve blocks (CPNBs) offer a number of benefits in the perioperative period, including flexibility in prolonging intraoperative analgesia [3], whilst avoiding the risks and side effects of opioids and general anaesthetics, such as postoperative nausea and vomiting [4,5], sedation, and respiratory depression [6]. In the postoperative period, CPNBs provide prolonged postoperative analgesia [7] and may contribute to faster postoperative rehabilitation [8].

Performing a continuous nerve or plexus block requires, after choosing a suitable site for catheter placement, a needle approach to the nerve or plexus using the in-plane or out-of-plane technique, hydrodissection, and catheter insertion [9]. Catheters currently used for continuous blocks are divided into two types, catheter-through-needle (CTN) and catheter-over-needle (CON), which differ slightly in their insertion technique [9]. Due to the fact that in the CTN method, the channel created by the needle in the tissues is slightly wider than the catheter itself [9], catheters of this type tend to leak and have a weaker fixation in the tissues than CON, which are firmly held by the surrounding tissues and do not exhibit a tendency for pericatheter leakage [10]. From the point of view of catheter design, there are single-orifice catheters, intended for peripheral nerve blocks, and multi-orifice catheters, which are more suitable for plexus blocks and interfascial blocks [11].

Continuous blocks, although generally safe, may fail because of catheter migration away from the intended tissue plane [12,13,14,15]. However, the present study was not designed to model real clinical catheter displacement, which is typically gradual, dynamic, partial, and potentially multidirectional. Instead, we used a simplified ex vivo comparison between two predefined catheter positions, interfascial and intramuscular, to determine whether measurable differences in normalized continuous infusion pressure can be observed under standardized low-rate infusion conditions. The aim of this pilot study was therefore exploratory and mechanistic rather than diagnostic and intended to inform whether further translational research is justified. The study does not evaluate detection, classification, or diagnostic performance in any form.

## 2. Materials and Methods

Before beginning the study, the protocol and the use of post mortem porcine tissues were reviewed by the Local Ethical Committee for Animal Experiments of the Medical University of Silesia in Katowice, Poland, reference BNW/NWN/0052/LKE/4/24, dated 26 April 2024. The Committee determined that formal approval was not required because the study involved only post mortem animal tissues and no live animals. The study utilised pig thighs. All pig thighs were obtained from “Przełom” Company, Bujaków, Poland. All specimens originated from male pigs with a body weight of approximately 120 kg. Because 60 thighs were included, the measurements were performed in batches of 10.

**Figure 1:**
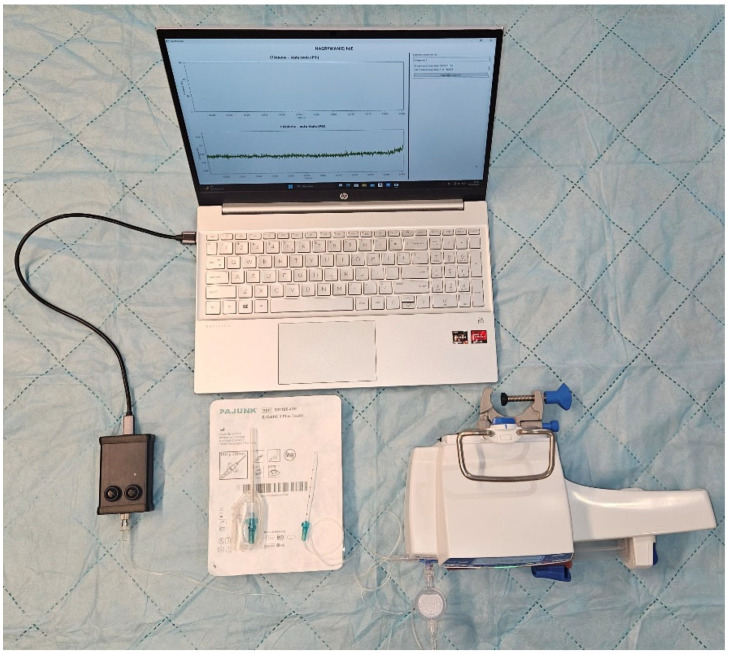
Measurement system.

Pajunk E-Cath^®^ II Plus Tsui^®^ 18G × 50 mm catheter-over-needle sets were used. This catheter-over-needle system features a multi-orifice catheter extending 15 mm beyond the plastic sleeve. A multi-orifice catheter was selected deliberately to reduce the likelihood that pressure measurements would be dominated by direct obstruction at a single distal opening by connective or muscle tissue, thereby allowing a more standardized comparison between the two predefined experimental positions. To minimize measurement artefacts, the tubing length was reduced to the absolute minimum. The measurement system (Figure 1) consisted of an Agilia SP TIVA pump with a dedicated 60 mL syringe, the Pajunk catheter set, and the InjectSense acquisition system incorporating a Honeywell ABP2LANG004BG2A3XX digital pressure sensor connected via USB to a laptop.

The pressure measurement system was based on a digital pressure sensor ABP2LANG004BG2A3XX (Honeywell ABP2 Series), designed for medical and industrial applications requiring stable and compensated pressure acquisition. The sensor is factory-calibrated and temperature-compensated using an integrated ASIC, which corrects for offset, sensitivity, non-linearity, hysteresis, and repeatability. According to the manufacturer’s specifications, the Total Error Band (TEB), representing the combined effect of all major error sources, is as low as ±1.5% of the full-scale span (FSS) [https://prod-edam.honeywell.com/content/dam/honeywell-edam/sps/siot/en-us/products/sensors/pressure-sensors/board-mount-pressure-sensors/basic-abp2-series/documents/sps-siot-abp2-series-datasheet-32350268-en.pdf (access on 10 April 2026)]. For the 4 bar measurement range used in this study, this corresponds to a maximum error of approximately ±0.06 bar (≈±0.87 PSI) over the full operating range. The nominal digital resolution of the sensor is approximately 0.0035 PSI.

The measurement chain consisted of the pressure sensor, a syringe pump, and short fluidic connections (tubing and catheter). The tubing used in the system has very low compliance, and under the low-pressure conditions investigated (on the order of a few PSI), its elastic deformation can be considered negligible. This assumption is consistent with commonly adopted simplifications in the literature. Consequently, the pressure measured at the sensor was assumed to approximate the effective pressure at the fluid outlet of the needle. This represents a modeling simplification that reduces system complexity while preserving comparability between experimental conditions.

To minimize measurement variability, all recordings were performed using an identical setup, fixed infusion rate, and controlled geometric configuration (constant vertical alignment of the sensor and infusion site). Additionally, signal processing included automated selection of a stable segment of the pressure trace, reducing the influence of transient fluctuations and observer-dependent bias.

Despite these measures, the measurement system was not subjected to a full independent metrological validation over the low-pressure range relevant to this study. Although comparative measurements were performed against a reference sensor (WIKA A-10), these do not constitute a formal calibration of the entire measurement chain. In particular, repeatability and reproducibility (intra- and inter-measurement variability) were not separately quantified.

An approximate signal-to-noise ratio (SNR) was estimated based on the observed between-group difference and within-group variability. The mean difference between groups was approximately 0.143 PSI, while the standard deviation ranged from 0.0285 to 0.0635 PSI. Using a conservative estimate based on the higher standard deviation, the SNR is approximately 2.25 (≈7 dB), and approximately 3.1 (≈10 dB) when using average variability. These values indicate that the observed signal exceeds typical variability but does not represent a high-margin separation from noise.

Importantly, this SNR estimate reflects combined variability from both measurement noise and biological or mechanical heterogeneity of the samples and therefore does not represent pure instrumental noise. Furthermore, manufacturer-specified error metrics apply to the full measurement range and may not directly reflect uncertainty in the narrow low-pressure regime studied here.

For these reasons, the measurement approach should be interpreted as suitable for controlled comparative analysis at the group level but not as a validated quantitative method for individual-level interpretation or clinical decision-making.

**Figure 2:**
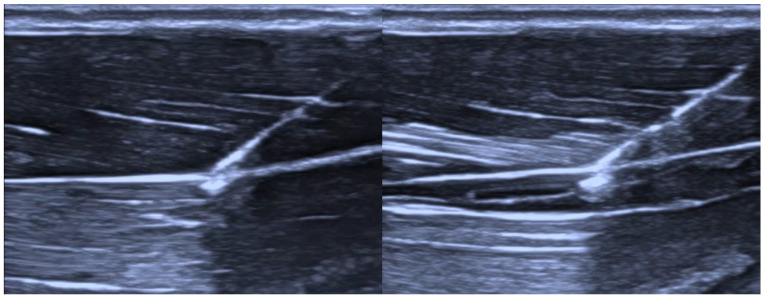
Puncture of the interfascial space (**left**) and its hydrodissection (**right**).

**Figure 3:**
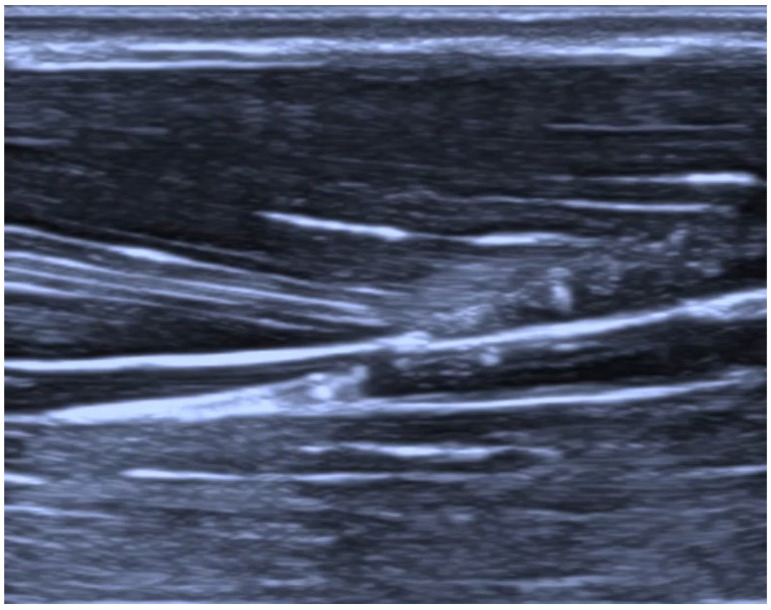
Nerve-block catheter in the interfascial space.

**Figure 4:**
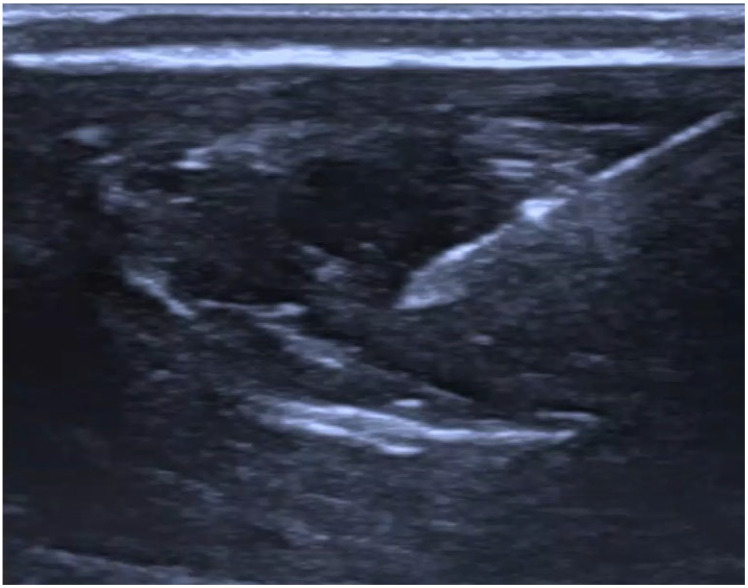
Intramuscular hydrodissection.

**Figure 5:**
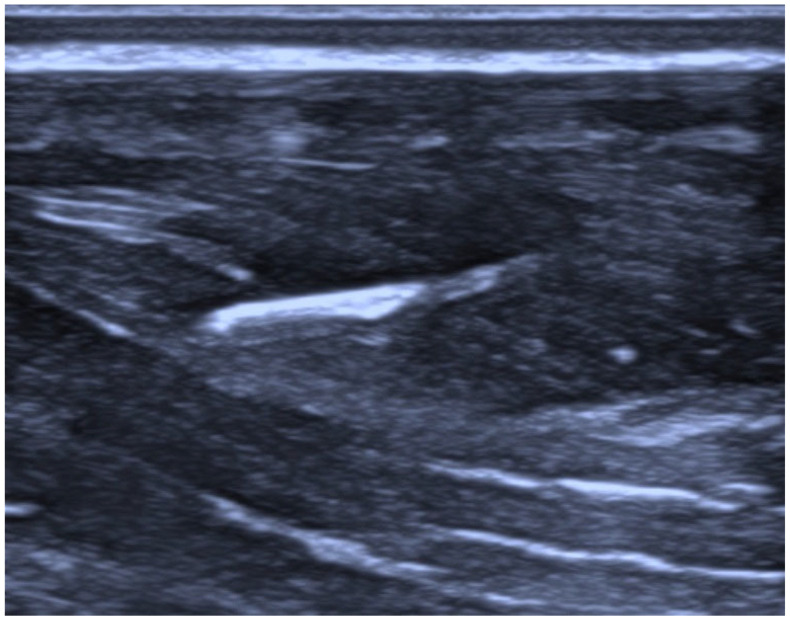
Nerve-block catheter in intramuscular location.

A baseline pressure measurement was first obtained during fluid delivery before catheter placement within the tissue. All measurements were performed with the pressure sensor and the injection site maintained at the same vertical level. Depending on the assigned experimental condition, the anesthesiologist inserted the catheter under ultrasound guidance either into the interfascial plane after hydrodissection (injection of saline into the tissue to create a space filled with it) with 2 mL of saline (Figure 2 and Figure 3) or intramuscularly after intramuscular saline hydrodissection (Figure 4 and Figure 5). The catheter assembly was then secured, and saline was infused at a fixed rate of 5 mL/h for 10 min. Each thigh was punctured only once and contributed one measurement only. Specimens were assigned alternately to one of two predefined experimental conditions, interfascial or intramuscular catheter placement. The final analysis included 30 specimens in the interfascial group and 30 specimens in the intramuscular group. Because allocation followed a predefined alternating sequence, no formal randomization procedure, sequence generation, or allocation concealment was used. The anesthesiologist performing the procedure was necessarily aware of the assigned placement condition but did not have access to the pressure measurement results during the procedure. Pressure is reported in PSI throughout the manuscript, with mmHg provided at first mention for clinical orientation. The collected data were then subjected to statistical analysis.

### Statistical Analysis

As stated above, baseline pressure was first measured during fluid delivery before catheter placement within the tissue, and all subsequent pressure recordings were normalized to this baseline. Because the pressure sensor and the injection site were maintained at the same vertical level and the same infusion system was used in all measurements, the reported normalized values should be interpreted as relative infusion pressure reflecting the additional flow resistance of the catheter–tissue environment under the specific experimental configuration rather than as an absolute tissue pressure measurement.

For each repetition in the interfascial and intramuscular conditions, a pressure time curve was recorded during a 10 min continuous infusion at a fixed rate of 5 mL/h. For each recording, the pressure time series was processed using a predefined automated pipeline. The raw signal was smoothed with a centered rolling median filter with a window of 11 points and subsequently with an exponentially weighted moving average with a span of 31 points. The most stable segment was defined as the longest contiguous interval of the filtered signal in which the total pressure range, defined as the difference between the maximum and minimum value within that interval, did not exceed a predefined threshold. For each recording, this threshold was set as the greater of 0.0090 PSI or 8% of the robust within-trace range, calculated as the difference between the 95th and 5th percentiles. Intervals shorter than 200 points were excluded. The representative value for each repetition was calculated as the mean of the filtered pressure values within the selected interval. All algorithm parameters were predefined before comparative analysis, and the procedure was applied identically to all recordings without manual segment selection or access to group labels.

Group comparisons were then performed using these representative mean normalized pressure values. Distributional assumptions were assessed with the Shapiro–Wilk test, and homogeneity of variance was evaluated with Levene’s test. Because the intramuscular group showed evidence of non-normality and group variances were unequal, between-group differences were tested using Welch’s two-sample *t*-test. Effect size was quantified using Hedges’ g as a standardized measure of between-group separation under the experimental conditions studied and not as a metric of diagnostic performance.

## 3. Results

**Figure 6:**
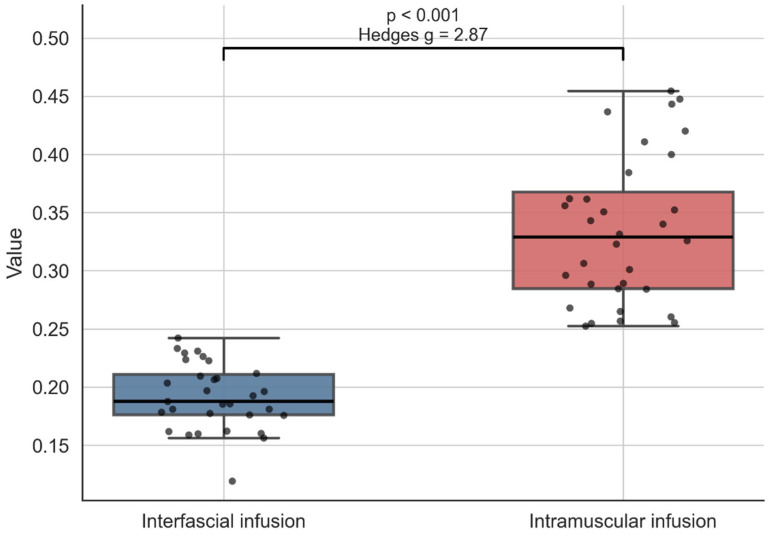
Boxplots of specimen-level representative normalized pressures in the interfascial and intramuscular groups. Each point represents one specimen and corresponds to the mean pressure calculated within the automatically identified stable segment of the filtered pressure trace.

The interfascial infusion group did not significantly deviate from normality (Shapiro–Wilk W = 0.9691, p = 0.4936), whereas the intramuscular infusion group showed evidence of non-normality (W = 0.9273, p = 0.0329). Variances differed significantly between groups (Levene’s test: F = 18.3026, p = 6.75 × 10^−5^). Mean normalized pressure was markedly higher in the intramuscular infusion group than in the interfascial infusion group (intramuscular infusion: mean = 0.3346, SD = 0.0635; interfascial infusion: mean = 0.1917, SD = 0.0285). Welch’s test confirmed a highly significant difference in mean pressure between groups, with higher values observed during intramuscular infusion, mean difference = 0.1430, t = 11.5990, p = 7.22 × 10^−15^, 95% CI: [0.1181, 0.1679]. The magnitude of this standardized between-group effect was very large (Hedges’ g = 2.8717); however, this reflects statistical separation between the two predefined experimental groups under controlled ex vivo conditions and does not imply clinical discriminative ability. Figure 6 displays all specimen-level representative values included in the analysis, with one point corresponding to one specimen.

## 4. Discussion and Conclusions

An analysis of the data obtained during the study reveals that the pressures generated in the interfascial space during continuous block are significantly lower, approximately 15–20 times, than those generated during single-shot blocks. This is most likely due to the much higher infusion rate in the latter [16,17].

A strength of the study is that the experimental setup was intentionally simplified and standardized in order to reduce avoidable procedural variability and allow a controlled comparison between two predefined tissue environments.

Several limitations must, however, be considered. First, this was a static ex vivo porcine model without perfusion, physiological tissue motion, or patient-specific anatomical variability, and post mortem porcine tissues differ from living human tissues in mechanical behaviour and compliance [18,19]. Second, the model compared two predefined catheter positions and was not intended to reproduce gradual, partial, multidirectional, movement-related, or delayed catheter migration in clinical practice. Third, each specimen contributed only one observation, so inter-specimen variability in tissue compliance, fat content, fascial integrity, and other specimen-specific properties were not controlled for. Fourth, allocation followed an alternating sequence rather than formal randomization, and operator blinding to placement condition was not feasible. Fifth, only one infusion setting was studied, namely saline at 5 mL/h for 10 min. We decided to use an infusion rate of 5 mL/h to meet the average flow rate used as the basal flow in clinical settings [9]. We tried to make this system as simple as possible to minimize the risk of measurement disturbances. As we did not study the aspects of bolus pressure or higher basal flow rates, further studies are needed. Consequently, the present data do not characterize the pressure-flow relationship and cannot be extrapolated to higher basal rates, bolus administration, longer recordings, drift over time, or delayed position change. What is also important is that pressure differences may scale with flow, and therefore, the current findings cannot be generalized to other infusion settings. Sixth, saline was used as a standardized experimental infusate, whereas clinical continuous blocks usually involve local anesthetic solutions, which have different physical properties than saline, like viscosity, tissue interactions and spread characteristics [20].

Finally, the pressure sensor is factory-calibrated according to manufacturer specifications. The observed mean between-group difference was 0.1430 PSI, which is substantially greater than the nominal digital resolution of the sensor, but it was not benchmarked against an independently validated full-system uncertainty estimate and therefore cannot be interpreted as establishing a clinically usable individual threshold. In addition, the representative value was derived from a predefined automated stable-segment algorithm. Although this procedure minimized observer-dependent selection bias, it may still influence the final estimate and should be regarded as an analysis strategy for this pilot study rather than a finalized clinical standard.

Accordingly, the present results should be interpreted only as exploratory and hypothesis-generating group-level observations. They demonstrate that a measurable difference in normalized infusion pressure can be observed between two predefined tissue environments under controlled ex vivo conditions, but they do not establish direct clinical usefulness, readiness for bedside application, or a validated individual diagnostic threshold. More clinically representative dynamic and in vivo studies are required before any translational interpretation can be considered.

This study showed a statistically significant between-group difference in normalized infusion pressure between the two predefined experimental positions (Figure 6 and Table 1). However, the absolute magnitude of this difference was small, which limits individual-level interpretability even within this controlled model. Similarly, the large Hedges’ g observed in this study should be understood only as a statistical descriptor of group-level separation within this simplified experimental model and should not be interpreted as evidence of clinical diagnostic utility or bedside discriminative performance. Importantly, even though the specimen-level representative values in the present dataset showed no overlap between groups, this should not be interpreted as evidence of reliable individual-level discrimination. The observed separation reflects this specific controlled ex vivo dataset and was not evaluated against full-system measurement uncertainty, robustness to added noise, or physiological variability expected in vivo. Therefore, no clinically usable individual threshold or diagnostic inference can be derived from the present results.

The present findings show that under standardized ex vivo conditions, mean normalized infusion pressure differs significantly between predefined interfascial and intramuscular catheter positions. However, the absolute difference is small. The model does not reproduce real clinical catheter displacement, the measurement methodology was not independently validated for clinical decision-making, and the findings were not evaluated against physiological variability present in vivo. Therefore, the results should be interpreted as exploratory and hypothesis-generating only. The study does not evaluate detection, classification, or diagnostic performance in any form. They support further translational research, but do not support current clinical applicability, individual-level interpretation, or the definition of a clinically usable threshold.

## Figures and Tables

**Table 1:** Basic statistics on continuous interfascial and intramuscular infusion pressures.

Statistics	Interfascial Infusion	Intramuscular Infusion
Mean [PSI]	0.1917	0.3347
Median [PSI]	0.1877	0.3288
Standard deviation	0.0280	0.0625
Min value [PSI]	0.1193	0.2526
Max value [PSI]	0.2421	0.4545

## Data Availability

Data are attached as Supplementary Materials in a separate file.

## Supplementary Materials

The following supporting information can be downloaded at: https://www.mdpi.com/article/10.3390/jcm15093301/s1.

## References

[1] Schultz P., Anker-Møller E., Dahl J.B., Christensen E.F., Spangsberg N., Faunø P. (1991). Postoperative pain treatment after open knee surgery: Continuous lumbar plexus block with bupivacaine versus epidural morphine. Reg. Anesth..

[2] Türker G., Uçkunkaya N., Yavaşçaoğlu B., Yilmazlar A., Ozçelik S. (2003). Comparison of the catheter-technique psoas compartment block and the epidural block for analgesia in partial hip replacement surgery. Acta Anaesthesiol. Scand..

[3] Borgeat A., Schäppi B., Biasca N., Gerber C. (1997). Patient-controlled analgesia after major shoulder surgery: Patient-controlled interscalene analgesia versus patient-controlled analgesia. Anesthesiology.

[4] Singelyn F.J., Aye F., Gouverneur J.M. (1997). Continuous popliteal sciatic nerve block: An original technique to provide postoperative analgesia after foot surgery. Anesth. Analg..

[5] Borgeat A., Tewes E., Biasca N., Gerber C. (1998). Patient-controlled interscalene analgesia with ropivacaine after major shoulder surgery: PCIA vs. PCA. Br. J. Anaesth..

[6] Singelyn F.J., Deyaert M., Joris D., Pendeville E., Gouverneur J.M. (1998). Effects of intravenous patient-controlled analgesia with morphine, continuous epidural analgesia, and continuous three-in-one block on postoperative pain and knee rehabilitation after unilateral total knee arthroplasty. Anesth. Analg..

[7] Chelly J.E., Greger J., Gebhard R., Coupe K., Clyburn T.A., Buckle R., Criswell A. (2001). Continuous femoral blocks improve recovery and outcome of patients undergoing total knee arthroplasty. J. Arthroplast..

[8] Capdevila X., Barthelet Y., Biboulet P., Ryckwaert Y., Rubenovitch J., d’Athis F. (1999). Effects of perioperative analgesic technique on the surgical outcome and duration of rehabilitation after major knee surgery. Anesthesiology.

[9] Maghami S., Ashken T., Lobo C. (2025). Peripheral nerve catheters for regional anaesthesia. BJA Educ..

[10] Kim H.Y., Ahn J.S., Park S., Choi E.J., Ri H.S., Yoon J.U., Byeon G.J. (2021). Comparison of catheter-over-needle and catheter-through-needle methods in ultrasound-guided continuous femoral nerve block: A prospective, randomized controlled trial. Medicine.

[11] Fegley A.J., Lerman J., Wissler R. (2008). Epidural multiorifice catheters function as single-orifice catheters: An in vitro study. Anesth. Analg..

[12] Schulz-Stübner S., Czaplik M. (2013). Qualitätsmanagement in der Regionalanästhesie am Beispiel des Regionalanästhesie Surveillance Systems (RASS) [Quality management in regional anesthesia using the example of a Regional Anesthesia Surveillance System (RASS)]. Der Schmerz.

[13] Marhofer D., Marhofer P., Triffterer L., Leonhardt M., Weber M., Zeitlinger M. (2013). Dislocation rates of perineural catheters: A volunteer study. Br. J. Anaesth..

[14] Steffel L., Howard S.K., Borg L., Mariano E.R., Leng J.C., Kim T.E. (2017). Randomized comparison of popliteal-sciatic perineural catheter tip migration and dislocation in a cadaver model using two catheter designs. Korean J. Anesthesiol..

[15] Fujino T., Yoshida T., Kawagoe I., Hinotsume A., Hiratsuka T., Nakamoto T. (2023). Migration rate of proximal adductor canal block catheters placed parallel versus perpendicular to the nerve after total knee arthroplasty: A randomized controlled study. Reg. Anesth. Pain Med..

[16] Roberto D., Quadri C., Capdevila X., Saporito A. (2023). Identification of interfascial plane using injection pressure monitoring at the needle tip during ultrasound-guided TAP block in cadavers. Reg. Anesth. Pain Med..

[17] Steinfeldt T., Quadri C., Saporito A., Musiari M., Capdevila X., Wiesmann T. (2020). Injection pressure monitoring at the needle tip for detection of perineural and nerve-contact position: A cadaver study. Can. J. Anaesth..

[18] Bishop J.H., Fox J.R., Maple R., Loretan C., Badger G.J., Henry S.M., Vizzard M.A., Langevin H.M. (2016). Ultrasound Evaluation of the Combined Effects of Thoracolumbar Fascia Injury and Movement Restriction in a Porcine Model. PLoS ONE.

[19] Trotta A., Ní Annaidh A. (2019). Mechanical characterisation of human and porcine scalp tissue at dynamic strain rates. J. Mech. Behav. Biomed. Mater..

[20] Istenič S., Pušnik L., Kenneth Ugwoke C., Pintarič T.S., Umek N. (2026). Mechanistic insights into bupivacaine spread through anisotropic tissue planes and fascial barriers: Experimental evidence for interfascial block dynamics. Reg. Anesth. Pain Med..

